# EBV finds a polycomb-mediated, epigenetic solution to the problem of oncogenic stress responses triggered by infection

**DOI:** 10.3389/fgene.2013.00212

**Published:** 2013-10-24

**Authors:** Martin J. Allday

**Affiliations:** Section of Virology, Department of Medicine, Imperial College LondonLondon, UK

**Keywords:** Epstein–Barr virus, PcG, epigenetic, oncogenic stress response, oncogene-induced senescence, p16^INK4a^, BIM, B cell transformation

## Abstract

Viruses that establish a persistent infection, involving intracellular latency, commonly stimulate cellular DNA synthesis and sometimes cell division early after infection. However, most cells of metazoans have evolved “fail-safe” responses that normally monitor unscheduled DNA synthesis and prevent cell proliferation when, for instance, cell proto-oncogenes are “activated” by mutation, amplification, or chromosomal rearrangements. These cell intrinsic defense mechanisms that reduce the risk of neoplasia and cancer are collectively called oncogenic stress responses (OSRs). Mechanisms include the activation of tumor suppressor genes and the so-called DNA damage response that together trigger pathways leading to cell cycle arrest (e.g., cell senescence) or complete elimination of cells (e.g., apoptosis). It is not surprising that viruses that can induce cellular DNA synthesis and cell division have the capacity to trigger OSR, nor is it surprising that these viruses have evolved countermeasures for inactivating or bypassing OSR. The main focus of this review is how the human tumor-associated Epstein–Barr virus manipulates the host polycomb group protein system to control – by epigenetic repression of transcription – key components of the OSR during the transformation of normal human B cells into permanent cell lines.

## INTRODUCTION – THE BIOLOGY OF EPSTEIN–BARR VIRUS (EBV)

Epstein–Barr virus (EBV) is a human gamma-herpesvirus (HHV4) and as such is characterized by a tropism for lymphocytes and an ability to persist life-long in the infected host. Data on persistent EBV infection in humans are consistent with the viral genome residing in a population of long-lived, largely non-dividing memory B cells. To establish persistence, EBV first infects resting (naïve) B cells – probably in tissues of the oropharynx – and transiently drives these to proliferate as activated B-blasts. The expanding B-blast population is thought to then either migrate into, or nucleate the formation of, a germinal center in local lymphoid tissue and therein the cells differentiate to become centroblasts, centrocytes, and finally resting memory B cells that enter the peripheral circulation (reviewed in Thorley-Lawson and Gross, 2004; Roughan and Thorley-Lawson, 2009). While the precise series of events that the EBV-positive B cells undergo to reach the memory compartment is not yet known, it is generally agreed that it involves regulated shut-down of latent EBV gene expression from an initial state called latency III, via latency II, until in quiescent memory B cells no EBV proteins can be detected in a state called latency 0. However, there is still some controversy as to whether or not the differentiation of EBV-infected B-blasts to resting memory B cells can occur anywhere outside the microenvironment of a germinal center (Rowe et al., 2009; Heath et al., 2012; Thorley-Lawson et al., 2013).

In more than 90% of the global population, following primary infection in infancy, EBV establishes an asymptomatic, stable, life-long, persistent infection in this long-lived pool of circulating memory cells. Periodic activation of an infected memory B cell by exposure to cognate antigen or aberrant T cell activity is thought to trigger plasma cell differentiation and concomitant “lytic” viral replication with the production of infectious virus that is released in the oropharynx and shed in saliva (reviewed in Thorley-Lawson and Gross, 2004; Laichalk and Thorley-Lawson, 2005; Roughan and Thorley-Lawson, 2009).

Primary EBV infection can cause the benign self-limiting disease infectious mononucleosis (IM) in some adolescents who were not infected in childhood. Uncontrolled proliferation of infected B cells in the immunocompromised of any age may result in a fatal form of IM, a chronic B lymphoproliferative disease or rarely the development of malignant immunoblastic lymphoma (Williams and Crawford, 2006). In normal individuals EBV-infected B-blasts are targets for EBV-specific cytotoxic T lymphocytes (CTLs) that recognize and destroy the EBV-infected proliferating B-blasts – so an equilibrium is established between B-blast proliferation on the one hand, and their immune-mediated elimination or differentiation to resting memory B cells on the other (Babcock et al., 1999; Thorley-Lawson and Gross, 2004; Hislop et al., 2007). Individuals who are co-infected with malaria or HIV are at increased risk of developing EBV-associated lymphomas, including Burkitt’s lymphoma (BL). EBV is also etiologically linked to subgroups of Hodgkin’s lymphoma (HL) and diffuse large B cell lymphoma (DLBCL), in addition to various non-B cell malignancies (reviewed in Young and Rickinson, 2004).

Infection of resting naïve B cells *ex vivo* with EBV can also induce the proliferation of the B-blast-like cells that *in vivo* would differentiate to become memory cells.* In vitro* these B cells do not differentiate, but are transformed to continuously proliferating permanent lymphoblastoid cell lines (LCLs) that retain the activated B cell phenotype and carry the viral genome as extra-chromosomal episomes. Only the nine latency III-associated proteins six nuclear (EBNAs 1, 2, 3A, 3B, 3C, and LP) and three membrane-associated (LMP1, LMP2A, and 2B) together with several RNA species are expressed from the viral genome (reviewed in Bornkamm and Hammerschmidt, 2001; Young and Rickinson, 2004; Forte and Luftig, 2011). These latency-associated gene products are responsible for activating the quiescent primary cells into the cell cycle, inducing and sustaining their proliferation and maintaining the extrachromosomal episome in these blast-like cells. There is general agreement – that at least in the initial stages after infection – LCL outgrowth recapitulates the early events of establishing latency prior to differentiation and long-term persistence *in vivo*. EBV may therefore be considered one of the few viruses that initiate and sustain the proliferation of infected cells as a necessary step in their life cycle, in the natural host. Some of the molecular details of how EBV does this in the face of intrinsic barriers to aberrant proliferation are the focus of this review. Specific attention will be paid to the polycomb group (PcG) protein-mediated epigenetic repression of the cyclin-dependent kinase inhibitor p16^INK4a^ and the pro-apoptotic BH3-only inducer of apoptosis BIM.

## ONCOGENIC STRESS RESPONSES (OSRs) AND ONCOGENE-INDUCED SENESCENCE (OIS)

**FIGURE 1:**
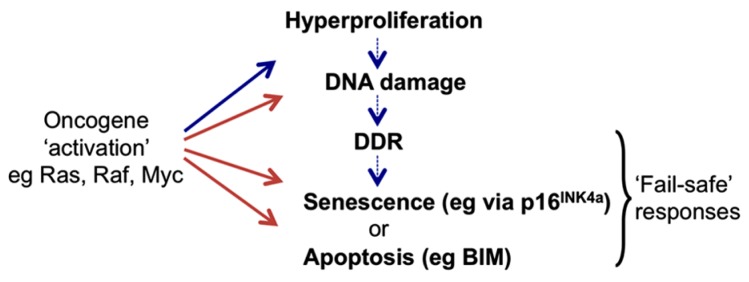
**Activation of cell proto-oncogenes can lead to oncogenic stress responses (OSR).** Oncogene “activation” by mutation or constitutive expression at supra-physiological levels can induce aberrant cell division that may become manifest as rapid cell proliferation (hyperproliferation). However higher vertebrates have evolved cell intrinsic “fail-safe” responses to recognize such cells and so block their proliferation or eliminate them completely. These can include the induction of tumor suppressors (ts) such as p16^INK4a^ that halt the cell cycle and can cause cells to enter a prolonged state of arrest called senescence, or pro-apoptotic proteins such as BIM that can induce programmed cell death (apoptosis). Responses may involve direct activation of the ts genes by oncoproteins or they can result from secondary signaling pathways (DDR) linked to the detection of damaged DNA produced during periods of aberrant DNA replication and cell division.

The seminal discovery in 1992 that the Myc proto-oncoprotein can trigger rapid apoptosis as well as cell growth and proliferation, led to the compelling hypothesis that apoptotic pathways must be disabled for oncogenes to promote neoplastic transformation of cells and the development of cancer (Askew et al., 1991; Evan et al., 1992). About 5 years later an equally influential discovery was that oncogenic mutant Ras protein – in addition to activating proliferative signaling pathways – also provokes in normal fibroblasts a cell cycle arrest resembling premature cell senescence. This was associated with the accumulation of tumor suppressors (ts) p53 and p16^INK4a^ (Serrano et al., 1997), and further endorsed the hypothesis that normal mammalian cells possess intrinsic defenses against oncogenic transformation. These observations inspired the concepts of “OSR,” “intrinsic tumor suppression,” and “OIS” and produced many detailed descriptions of mechanisms involving the p53 and retinoblastoma (Rb) tumor suppressor pathways that prevent deregulated oncogenes causing cancer (**Figure 1**; reviewed in Sherr, 1998,^2012^; Lowe et al., 2004; Braig and Schmitt, 2006).

## RELATIONSHIP BETWEEN OSR/OIS AND DNA DAMAGE RESPONSES (DDRs)

Since cell proto-oncogenes generally control signaling pathways and/or gene networks that link proliferative signals to the cell cycle machinery, when they are deregulated this can result in unscheduled entry into S phase and aberrant DNA synthesis (sometimes referred to as “replicative stress”). As a consequence, oncogene activation can produce the stalling of DNA replication forks that results in damaged DNA – particularly double strand breaks. Such lesions can also be caused by the action of multiple physical and chemical agents and they can trigger, primarily via the ATM/ATR-kinase signaling pathway, the stabilization and activation of p53 and also the induction of 16^INK4a^. Depending on the physiological and cellular context this leads to DNA repair, cell death, or senescence. This complex response is known as the DDR. It has been proposed that the induction of apoptosis or cell cycle arrest/senescence by oncogenic stress is a general downstream manifestation of the DDR acting as a barrier to cell transformation *in vitro* and tumor progression *in vivo* (Di Micco et al., 2006; Bartek et al., 2007; Halazonetis et al., 2008). However, it remains unclear whether all oncogene-mediated stress responses act via the DDR, or whether alternative signaling pathways directly regulate downstream effectors (see for example induction of p16^INK4a^ in response to oncogenic RAS/RAF signaling (Agger et al., 2009; Barradas et al., 2009) and the relationship between MYC and BIM in B cell lymphomas described below). The links between DDR, OSR, and OIS have been extensively reviewed (Braig and Schmitt, 2006; Gil and Peters, 2006; Kim and Sharpless, 2006; Wade and Wahl, 2006; Bartek et al., 2007; Halazonetis et al., 2008).

A common feature of herpesviruses is their capacity to activate DDRs in infected cells (Shirata et al., 2005; Gaspar and Shenk, 2006; Koopal et al., 2007; Tarakanova et al., 2007; Nikitin et al., 2010). Although in some cases this is associated with lytic or productive infection, when the virus has a requirement for rapid replication of its genome prior to virion assembly, at least two gamma-herpesviruses (Kaposi’s Sarcoma associated herpes virus (KSHV, aka HHV8) and EBV) trigger DDRs during the establishment of a latent infection. This is largely because latency-associated viral proteins drive cells into the cell cycle and can induce hyperproliferation, replication errors, and associated DNA damage (Koopal et al., 2007; Nikitin et al., 2010). Moreover, it has been suggested that EBV infection of B cells *in vitro* may also induce reactive oxygen species (ROS) that can damage DNA (reviewed in Allday, 2009; Gruhne et al., 2009). EBV and KSHV appear to have evolved mechanisms for the attenuation of the DDR to ensure latent infection is maintained. Virus-associated responses involving the DDR have recently been comprehensively reviewed elsewhere (Leidal et al., 2012; Nikitin and Luftig, 2012) and for EBV will be reconsidered below.

## THE *INK4b*-*ARF*-*INK4a* LOCUS, p16^INK4a^, OSR/OIS, AGING, AND CANCER

**FIGURE 2:**
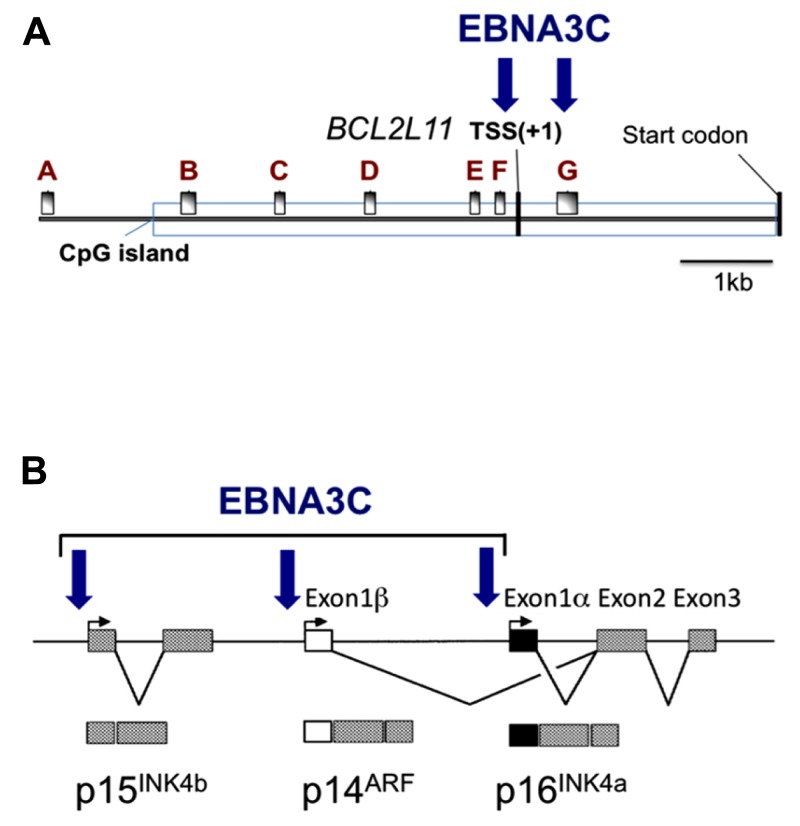
**Epitope-tagged EBNA3C associates with the promoter for BIM and genes in the *INK4b-ARF-INK4a* locus.** Schematic representations of **(A)** approximately 9kb of the *BCL2L11* (BIM) promoter and **(B)** approximately 40 kb including the *INK4b-ARF-INK4a* locus. Vertical arrows indicate the positions where EBNA3C has been detected by chromatin immunoprecipitation (ChIP) analyses using lymphoblastoid cell lines (LCL) established using EBV expressing an epitope-tagged EBNA3C. These same regions of chromatin are marked by the polycomb (PRC2)-mediated modification H3K27me3 when EBNA3A and EBNA3C are present (adapted from Paschos et al., 2012; Skalska et al., 2013). The *BCL2L11* gene transcriptional start site (TSS) and the protein products of *INK4b-ARF-INK4a* are indicated*.* A–G in **(A)** mark the positions of RT-PCR primer sets used in (Paschos et al., 2012).

Within the *INK4b-ARF-INK4a* locus at human chromosome 9p21, *CDKN2A* encodes two potent tumor suppressors, p16^INK4a^, and p14^ARF^ (p19^ARF^ in mice); these proteins are critical negative regulators of cell proliferation. Although exons 2 and 3 are shared by *INK4a* and *ARF*,** the proteins result from differential splicing and are encoded in alternative reading frames (reviewed in Gil and Peters, 2006; Kim and Sharpless, 2006; Sherr, 2012). Adjacent to *CDKN2A* is a second related gene *CDKN2B* that encodes a protein closely related to p16^INK4a^ called p15^INK4b^ (**Figure 2**). The cyclin-dependent kinase (CDK) inhibitor p16^INK4a^ acts on the cyclin D-dependent kinases (CDK4 and CDK6) abrogating their binding to D-type cyclins and so inhibiting CDK4/6-mediated phosphorylation of the Rb protein. By binding CDKs and blocking Rb hyperphosphorylation, increased p16^INK4a^ expression causes a G1 cell cycle arrest and senescence (Gil and Peters, 2006; Kim and Sharpless, 2006; Sherr, 2012). Although the CDK inhibitor p15^INK4b^ has about 85% amino acid similarity to p16^INK4a^ and biochemically behaves in much the same way, in most mammalian cells – for unknown reasons – it has distinct functions. In contrast to the CDK inhibitors, the p14 and p19 ARF proteins regulate the stability of p53 by inactivating MDM2 – a p53-specific ubiquitin ligase that facilitates p53 degradation. The concomitant stabilization and activation of p53 leads to G1 and G2 cell cycle arrest by inducing the CDK regulator p21^WAF1^ or apoptosis by inducing pro-apoptotic factors such as NOXA and PUMA (Vousden and Prives, 2009; Sherr, 2012).

The products of *CDKN2A* can therefore be key mediators of OSR and potent barriers to the “immortalization” of cells in culture and the development of cancers *in vivo*. Both p16^INK4a^ and ARF are also progressively up-regulated with tissue aging, when they probably contribute to the aging process by reducing reservoirs of stem cells capable of self-renewal (Kim and Sharpless, 2006; Collado et al., 2007). There is general agreement that p19^ARF^ plays the more important role in all these processes in mice, whereas in human cells p16^INK4a^ is the dominant player. It is therefore not surprising that in a wide variety of human cancers *INK4a* is inactivated by gene deletion, mutation, or promoter DNA methylation (Gil and Peters, 2006; Kim and Sharpless, 2006; Popov and Gil, 2010). The whole* INK4b-ARF-INK4a* locus appears to be coordinately regulated epigenetically by polycomb protein complexes generating repressive histone modifications (Gil and Peters, 2006; Popov and Gil, 2010). Although induction of p16^INK4A^ in fibroblasts and epithelial cells is generally associated with cell cycle arrest and senescence, in B cells – which exhibit no obvious characteristics of senescence – there may be some crosstalk between p16^INK4a^ and the apoptotic machinery, since in lymphocytes the default pathway triggered by p16^INK4a^ can be death rather than prolonged cell cycle arrest (Lagresle et al., 2002; Bianchi et al., 2006).

## BIM, B CELLS, AND MYC

BIM (*B*cl2-*i*nteracting *m*ediator) is a pro-apoptotic member of the BH3-only family of BCL2-like proteins and is encoded by the *BCL2L11* gene at human chromosome 2q13. BIM acts as a potent, direct initiator of apoptosis because it binds with high affinity to BCL2 and all the other pro-survival family members to inactivate them. BIM also binds and activates pro-apoptotic BAX to initiate cytochrome-c release from mitochondria (Strasser, 2005; Gavathiotis et al., 2008). BIM is particularly important in the immune system, acting as a major regulator of life-and-death decisions during lymphocyte development including the negative selection of auto-reactive B cells and programmed death of low-affinity antibody-expressing germinal center-derived B cells (Enders et al., 2003; Strasser, 2005; Fischer et al., 2007). *Bim*-null mice accumulate excess lymphoid and myeloid cells and loss of *Bim* accelerates B cell lymphomagenesis induced by an *Eμ*-*Myc* transgene. Even loss of a single allele accelerates lymphomagenesis significantly, indicating *Bim* is a haploinsufficient tumor suppressor and that the level of Bim protein is rate-limiting in murine B cell survival (Egle et al., 2004).

Extending this *Eμ*-*Myc*-lymphoma model to human B cell lymphomagenesis, the relationship between *MYC* and BIM in EBV-negative BL was investigated (Dang et al., 2005; Hemann et al., 2005). This study brought into sharp focus the activation of *BCL2L11/BIM* by MYC, and led to the proposal that *MYC*-induced apoptosis can be overridden by inactivation of any one of several *MYC* effectors – including p53, p14^ARF^, or BIM – causing apoptosis-firing to drop below a critical threshold to allow cell proliferation. It also established that *BCL2L11/BIM* is a p14^ARF^/p53-independent target of MYC and that its activation does not require MYC-induced hyperproliferation (Dang et al., 2005; Hemann et al., 2005). Thus BIM is a uniquely important tumor suppressor in cells of the hematopoietic lineage and operationally its activation by MYC is a component of the OSR in B cells. Since MYC is induced and becomes constitutively expressed early after EBV infection of primary human B cells, modulation of BIM expression by EBV is likely to be a contributory factor in B cell transformation and the development of any EBV-associated B cell lymphomas (discussed in more detail below).

## POLYCOMB GROUP PROTEINS AND EPIGENETIC REPRESSION

Epigenetic gene regulation is heritable and results from changes in a chromosome without alterations to DNA sequence (Berger et al., 2009). Such changes can be mediated by chemical modifications to chromatin on either DNA or DNA-associated histones and may involve non-coding RNAs. PcG proteins were first identified in *Drosophila* and are best known as repressors of the homeotic (Hox) transcription factor genes during embryonic development. They are very highly conserved from flies to humans and homologues regulating developmental transitions are found in plants. PcG proteins form multi-protein complexes called polycomb repressive complexes (PRCs) that bind and epigenetically regulate hundreds of genes, predominantly associated with cell-fate decisions and development (reviewed in Bracken and Helin, 2009; Margueron and Reinberg, 2011; Bemer and Grossniklaus, 2012; Simon and Kingston, 2013). They can repress transcription by introducing post-translational covalent modifications on histones in chromatin located in the regulatory regions of target genes. This repression/silencing is stable and heritable so can be described as epigenetic (Berger et al., 2009).

PRC2 is a multi-component complex that mediates tri-methylation at lysine 27 of histone H3 (H3K27me3). In humans the core complex is comprised of three polycomb proteins: suppressor of zeste (SUZ)12, embryonic ectoderm development (EED), and enhancer of zeste (EZH)2. EZH2 contains the catalytic SET domain responsible for lysine methyltransferase activity (Bracken and Helin, 2009; Margueron and Reinberg, 2011; Simon and Kingston, 2013). Other components of PRC2 are histone chaperone RbAp46/48 and recently an ancillary factor, JARID2, has been identified as being essential for recruitment of PRC2 to some polycomb-target genes (Murzina et al., 2008; Landeira et al., 2010; Margueron and Reinberg, 2011; Simon and Kingston, 2013). It remains unclear how in most cases the polycomb proteins are recruited to specific promoters in mammalian cells, although sequence context is probably important and a preference for regions rich in CpG dinucleotides (CpG-islands) has been reported (Ku et al., 2008). However, for most target genes, it remains to be determined whether specificity comes from sequence-specific transcription factors, PRC2-interacting non-coding RNA species, or yet to be identified mechanisms (Bracken and Helin, 2009; Khalil et al., 2009; Gupta et al., 2010; Kanhere et al., 2010; Simon and Kingston, 2013).

H3K27me3 on chromatin attracts the binding of a second complex, PRC1 that mediates the repressive ubiquitinylation at lysine 119 of histone H2A (H2AK119Ub). PRC1 core proteins include chromobox (CBX) proteins, whose chromodomains are thought to recruit the complex to the H3K27me3 mark, and RING finger proteins, such as RING1B, MEL18, and BMI1 that are responsible for the E3 ubiquitin ligase activity that produces H2AK119Ub. PRC1 mediates chromatin compaction and the local formation of heterochromatin (Grau et al., 2011) and together with PRC2, increases the chances of the more stable CpG DNA methylation mark being deposited (reviewed in Cedar and Bergman, 2009). Although recent evidence suggests H3K27me3 is stable and heritable (Simon and Kingston, 2013) this histone modification can be rapidly removed by demethylase enzymes such as JMJD3 (aka KDM6B; Agger et al., 2009; Barradas et al., 2009). Moreover, if a promoter carries H3K27me3 and simultaneously has the activation-associated modification H3K4me3 at the same locus, it is repressed but is described as “bivalent” and thought to be poised for rapid reactivation by removal of H3K27me3; genes with such bivalent domains are common in stem cells (Bernstein et al., 2006; Voigt et al., 2013). Cancer cells and stem cells often share gene expression patterns and multiple reports suggest that polycomb complexes contribute to the aberrant CpG DNA methylation profiles that are critical in the genesis and progression of many diverse cancers (Cedar and Bergman, 2009). The mechanism for this is suggested by the capacity of various polycomb proteins to physically interact with DNA methyl transferases (DNMTs) and recruit them to chromatin. It has been estimated that PcG-target genes are up to 12 times more likely to be aberrantly methylated in cancer than non-targets (Widschwendter et al., 2007).

## EPIGENETIC REGULATION OF BIM AND p16^INK4a^ EXPRESSION BY EBV

### EBNA3A AND EBNA3C COOPERATE AS ONCOGENIC REPRESSORS OF TRANSCRIPTION

The EBV EBNA3 proteins (EBNA3A, EBNA3B, and EBNA3C) are large (>900 aa) latency-associated nuclear proteins that show no significant similarity to known cell or viral factors. Although none of them appears to bind DNA directly, they all bind the cellular DNA-binding factor CBF-1 (aka RBP-JK; reviewed in Bornkamm and Hammerschmidt, 2001; Young and Rickinson, 2004). All three EBNA3s can also interact with cellular factors associated with the covalent modification of histones, the repression of transcription, and gene silencing; for example, EBNA3A and EBNA3C associate with histone deacetylases (HDACs) and the conserved co-repressor CtBP (Radkov et al., 1999; Bornkamm and Hammerschmidt, 2001; Touitou et al., 2001; Hickabottom et al., 2002; Young and Rickinson, 2004). EBNA3A, EBNA3B, and EBNA3C are all robust repressors of transcription when targeted directly to DNA in transient assays (Bain et al., 1996; Cludts and Farrell, 1998 and our unpublished data), and EBNA3A and EBNA3C – but not EBNA3B – are necessary to establish LCLs from purified B cells (Tomkinson and Kieff, 1992; Tomkinson et al., 1993). EBNA3A and EBNA3C also cooperate with oncogenic Ha-Ras in the transformation/immortalization of primary rodent fibroblasts and require the interaction with CtBP to do this (Parker et al., 1996; Touitou et al., 2001; Hickabottom et al., 2002). All the data are therefore consistent with EBNA3A and EBNA3C acting as oncoproteins in the transformation of B cells and in EBV-associated lymphomagenesis. However EBNA3B is unnecessary in these processes, and can even act as a tumor suppressor (White et al., 2012).

Recent microarray gene-expression analyses using LCLs or lymphoma cells infected with recombinant B95.8 strain EBVs that express defined EBNA3 mutants, suggest that together the EBNA3s can regulate >1000 host genes in B cells – often repressing transcription. The regulation of many of these genes seems to require the functional interaction of at least two EBNA3s and in several cases that have been subjected to further analysis, gene repression appears to utilize the host PcG system to inhibit transcription via the H3K27me3 chromatin modification (Hertle et al., 2009; Skalska et al., 2010; White et al., 2010,^2012^; Maruo et al., 2011; Zhao et al., 2011; McClellan et al., 2012; Paschos et al., 2012). Two genes repressed by the combined action of EBNA3C, EBNA3A, and PcG proteins – and of particular interest in the context of OSR – encode BIM and p16^INK4a^.

### REPRESSION OF BIM TRANSCRIPTION

**FIGURE 3:**
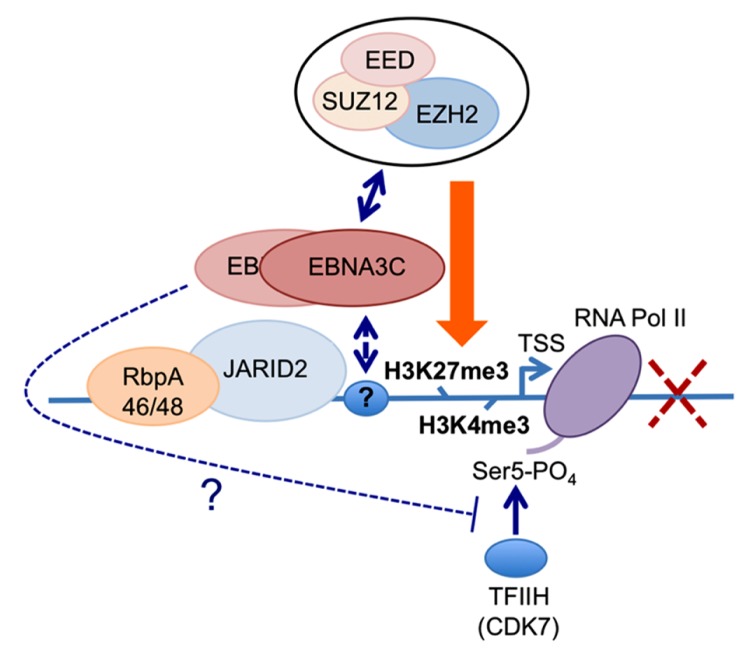
**Working hypothesis for the role(s) of EBNA3C and EBNA3A in the PRC2-mediated repression of the BIM promoter.** The available data indicate that EBNA3C (and EBNA3A) are recruited to regions proximal to the *BCL2L11/BIM* transcriptional start site (TSS) in EBV-infected B cells (Paschos et al., 2012; our unpublished data and **Figure 2**). Irrespective of whether EBNA3C or EBNA3A are expressed in these cells, the PRC2-associated factors RbpA46/48 and JARID2 are present at the locus. Similarly the activation mark H3K4me3 and RNA polymerase II (RNA Pol II) occupy the TSS irrespective of which EBNA3s are expressed. Only when both EBNA3C and EBNA3A are present are core components of the PRC2 complex found at this site and the repressive chromatin mark H3K27me3 is detected across the TSS; concomitantly the level of transcription and BIM expression are reduced. The simultaneous presence of both H3K4me3 and H3K27me3 at the locus define it as a “bivalent” or “poised” domain and is consistent with RNA Pol II always being detected. However only in the absence of either EBNA3C or EBNA3A is RNA Pol II phosphorylated on serine residue 5 (RNA Pol II Ser 5), suggesting that in addition to playing a key role in the recruitment of PRC2 core complex, the presence of EBNA3C and EBNA3A might interfere with serine 5 phosphorylation of RNA Pol II and therefore block the initiation of transcription. Since EBNA3A and EBNA3C can be co-immunoprecipitated from infected B cells and both are necessary for repression of BIM (and p16^INK4a^) expression, in this model we assume they are co-localized at these loci. The identity of the factor(s) responsible for targeting EBNA3C and/or EBNA3A to this particular stretch of chromatin is still unknown, as is the mechanism of interaction with PRC2.

The first indication that EBNA3A and EBNA3C can cooperate to repress specific host cell genes came using a panel of EBNA3-knockout recombinant B95.8-derived EBVs to infect EBV-negative BL cells. This revealed that expression of both EBNA3A and EBNA3C are necessary to repress transcription of *BCL2L11/BIM* (Anderton et al., 2008). Subsequently it was found that DNA in a large CpG island located at the 5′ end of *BCL2L11/BIM* becomes methylated on CpG dinucleotides in EBV-positive BLs (Paschos et al., 2009). However a reduction in BIM expression occurred soon after EBV infection of B cells in culture and did not initially involve detectable CpG methylation, but correlated with the deposition of the polycomb signature H3K27me3 on chromatin proximal to the transcription start site (TSS; Paschos et al., 2009,^2012^). Detailed chromatin immunoprecipitation (ChIP) analyses of the chromatin around the *BCL2L11/BIM* promoter revealed that latent EBV triggers the recruitment of polycomb repressive complex 2 (PRC2) core subunits and the trimethylation of histone H3 lysine 27 (H3K27me3) at this locus. It appears that in uninfected BL cells, RbAp48, and JARID2 already associate with the chromatin proximal to the TSS and that EBV infection is necessary to recruit SUZ12 and EZH2 to establish functional PRC2. Assembly of PRC2 at the locus was absolutely dependent on both EBNA3A and EBNA3C being expressed, and using a recombinant EBV expressing an epitope-tagged EBNA3C, it was shown by ChIP that EBNA3C associates with chromatin near the TSS – it is therefore likely to physically interact with PRC2 (Paschos et al., 2012; **Figure 2** and model in **Figure 3**). Since the activation mark H3K4me3 is largely unaltered at this locus irrespective of H3K27me3- or EBNA3-status the establishment of a “bivalent” chromatin domain is suggested. Consistent with the “poised” nature of these domains, RNA polymerase II (RNA Pol II) occupancy at the *BCL2L11/BIM* TSS was not altered by EBV. However, further analysis of phospho-serine 5 on RNA Pol II indicated that when EBNA3A and EBNA3C are both expressed they inhibit this phosphorylation step and block the initiation of the *BIM* transcripts. It was not determined whether this involves the direct action of an EBV protein on the kinase CDK7 or is a consequence of the recruitment of PRC2 and/or PRC1 to this particular locus. B cell lines carrying EBV encoding a conditional EBNA3C-modified estrogen receptor-fusion revealed that this epigenetic repression of* BIM* was reversible, but took more than 30 days from when EBNA3C was inactivated, emphasizing the stability of these chromatin modifications through rounds of cell division. Lentivirus delivery of shRNAs against PRC2 and PRC1 subunits disrupted EBV repression of *BCL2L11/BIM,* thus confirming the requirement for PcG complexes (Paschos et al., 2012).

### REPRESSION OF TRANSCRIPTION FROM THE CDKN2A LOCUS

Direct evidence that EBNA3C modulates the cell cycle during EBV-mediated transformation of B cells came from Maruo et al. (2006). Using a recombinant Akata strain EBV made conditional for EBNA3C function by fusing EBNA3C with a modified estrogen receptor, they revealed that EBNA3C represses expression of the CDK inhibitor p16^INK4A^ in LCLs. Removing the inducer of EBNA3C activity (4-hydroxytamoxifen, 4HT) from the culture medium resulted in an accumulation of both p16^INK4A^ mRNA and protein, de-phosphorylation of Rb, and concomitant cell cycle arrest (Maruo et al., 2006). Using a similar recombinant virus expressing an EBNA3A-fusion, the same authors showed that inactivation of EBNA3A also resulted in reduced proliferation, although the mechanism was not determined (Maruo et al., 2003). Since EBNA3A and EBNA3C are necessary for the H3K27me3-mediated chromatin manipulation and epigenetic repression of *BCL2L11/BIM*, and since the *CDKN2A* locus that encodes p16^INK4a^ had been identified as a target of polycomb-mediated repression in proliferating cells, it was not surprising to discover that the combined action of EBNA3C and EBNA3A repressed *CDKN2A* in cycling B cells by facilitating the deposition of H3K27me3 across the locus – primarily around the p16^INK4a^ TSS (Skalska et al., 2010). Furthermore, establishing LCLs with recombinant viruses encoding CtBP-binding mutants of EBNA3C and EBNA3A revealed that their interaction with this highly conserved cellular co-repressor was necessary for the efficient deposition of H3K27me3 and repression of p16^INK4a^ expression. ChIP analysis for the epitope-tagged EBNA3C expressed in an LCL revealed EBNA3C at the TSS of p16^INK4A^ and ARF, and also the *CDKN2B* gene encoding p15^INK4b^ (**Figure 2**; Skalska et al., 2013). Although it was initially unclear whether the EBNA3C-associated H3K27me3 deposition at *CDKN2A* was a cause or a consequence of cells exiting from the cell cycle, regulation of the locus by EBNA3C in an Rb-null LCL (Skalska et al., 2010) and in several p16^INK4a^-null LCLs (Skalska et al., 2013 and see below) unequivocally established that EBNA3C regulation of the locus is independent of the degree of cell proliferation. As with *BLC2L11/BIM*, B cell lines carrying EBV encoding the conditional EBNA3C-modified estrogen receptor-fusion revealed that this epigenetic repression of* CDKN2A* was reversible by adding or removing 4HT from the medium. Taken together all these data suggest that EBNA3C (cooperating with EBNA3A) coordinately regulates the whole *INK4b-ARF-INK4a* locus by directing the recruitment of PRC2 to the three transcriptional start sites. Consistent with this we have recently found that the level of p15^INK4b^ mRNA is coordinately regulated with that of p16^INK4a^ in EBNA3C-conditional LCLs (our unpublished data).

The specific role of p16^INK4a^ as a target for EBNA3C and a major barrier to B cell transformation was further explored making use of an “experiment of nature” in the form of “Leiden” B cells carrying a homozygous genomic deletion that specifically ablates production of functional p16^INK4a^ (Brookes et al., 2002; Hayes et al., 2004). These cells were infected with recombinant B95.8-derived EBVs that express either the conditional EBNA3C or no EBNA3C (Skalska et al., 2013). A comparison of p16-null LCLs with LCLs established from normal B cells showed unequivocally that, if p16^INK4a^ is not functional, then EBNA3C is unnecessary to sustain cell proliferation. Consistent with this – and providing formal proof that p16^INK4a^ is the main target of EBNA3C – it was possible to transform p16-null B cells into stable LCLs with EBV, but without any functional EBNA3C ever having been expressed.

### INHIBITING OSR/OIS IS NECESSARY FOR LCL OUTGROWTH

**FIGURE 4:**
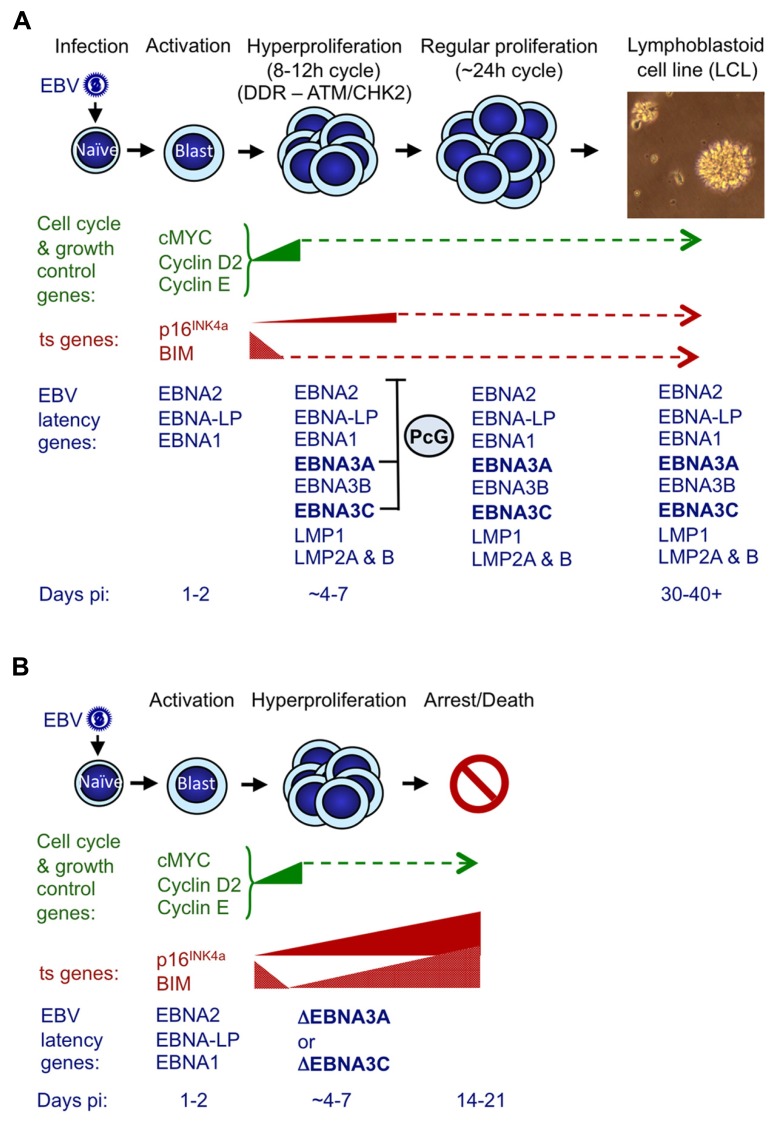
**Events following infection of primary resting B cells by EBV that initiate transformation into continuously proliferating LCLs.** **(A)** During the first 24–48 h post-infection (pi) with a B95.8-derived EBV, cell genes associated with growth and cell cycle are transactivated and their products (e.g., MYC, cyclin D2, cyclin E) drive cells from G0 to G1, to become enlarged, activated and start proliferating. The whole process is driven by the EBV transactivator protein EBNA2, probably assisted by the co-factor EBNA-LP (Sinclair et al., 1994; Spender et al., 1999; Nikitin et al., 2010). During the next 3–4 days cells undergo rounds of rapid cell division (hyperproliferation) and in some cells this results in damage to DNA that can activate the DNA damage response (DDR) and initiate a signaling cascade involving the kinases ATM and CHK2 (Nikitin et al., 2010). If the full complement of nine EBV latency-associated proteins is expressed, the DDR becomes attenuated (in part by EBNA3C) and cells continue to proliferate to produce polyclonal LCLs that have a population doubling time of about 24 h. Early after infection BIM expression is down-regulated, and although the level of p16^INK4a^ expression increases slightly, this soon reaches a steady state. In both cases we assume that EBNA3A and EBNA3C cooperate by harnessing the polycomb group (PcG) protein system to epigenetically repress (or restrain the transcription of) these ts genes via H3K27me3 (Anderton et al., 2008; Paschos et al., 2012; Skalska et al., 2013). **(B)** If EBNA3C or EBNA3A are deleted (ΔEBNA3C and ΔEBNA3A) or functionally inactivated in the infecting EBV, beginning about 4–7 days pi, the levels of mRNAs corresponding to p16^INK4a^ and BIM progressively increase and continue to do so for the next week or two until finally most of the cells arrest and/or die (Skalska et al., 2013 and our unpublished data). The PcG-mediated repression of these two ts genes – in particular p16^INK4a^ (see text) – is part of a critical countermeasure evolved by EBV to bypass an intrinsic host defense against oncogenic transformation. If primary B cells are p16^INK4a^-null, functional EBNA3C is dispensable for the outgrowth of LCLs. This is consistent with p16^INK4a^ being the dominant barrier to outgrowth and subsequent proliferation of LCLs, and the principal requirement of EBNA3C appears to be to restraining transcription of p16^INK4a^ (see text for details and Skalska et al., 2013). The precise relationships between DDR, p16^INK4a^ and EBNA3C/EBNA3A have yet to be defined.

A reasonable but speculative explanation for why EBV has evolved a mechanism for suppressing p16^INK4a^ (and BIM) expression became apparent from examining the outcome of attempted transformations of normal B cells with EBNA3C-deficient EBV (**Figure 4**; Skalska et al., 2013). These experiments revealed that infection with a “wild type” EBV modestly induced p16^INK4a^ transcription in the first few days after infection – when EBNA2 transactivates inducers of cell cycle progression (e.g., MYC and cyclin D2) and a period of hyperproliferation has been described (Sinclair et al., 1994; Spender et al., 1999; Nikitin et al., 2010). It is likely that unscheduled entry into S-phase, is interpreted by the cell as oncogenic stress and activation of p16^INK4a^ transcription is a consequence. When the infecting virus expressed functional EBNA3C (and EBNA3A) there was a halt to the increase of p16^INK4a^ expression from about day 7 onwards. However, if EBNA3C was not expressed or was non-functional (i.e., no 4HT in the medium), transcription from *INK4a* continued unrestrained and the level of mRNA progressively increased over the next 2–3 weeks, until most of the cells stopped proliferating. Early after infection BIM expression is down-regulated, and very soon (<5 days) reaches a steady state, but if EBNA3C is deleted or functionally inactivated in the infecting EBV – beginning about 4 days post infection – the level of mRNA corresponding to BIM also increases, in parallel with that of p16^INK4a^. This increase continues for the next week or two until cells arrest or die (Skalska et al., 2013). Largely similar results were obtained with EBNA3A-negative virus (our unpublished data).

The EBNA3C/3A-mediated epigenetic inhibition of *INK4a* and *BCL2L11/BIM* transcription is therefore critical for EBV to bypass an intrinsic host cell defense against oncogenic transformation probably triggered by EBNA2 acting through MYC (summarized in **Figure 4**; see also Nikitin et al., 2010). Thus expression of both EBNA3C and EBNA3A ensures expansion of the infected B cell population and LCL outgrowth *in vitro* and *in vivo* the initiation of latency. Strictly speaking, in this context, EBNA3C and EBNA3A do not actually repress *INK4a* and* BCL2L11/BIM* transcription, but rather prevent their activation. This most likely involves the recruitment of PcG protein complexes to the loci, leading to H3K27me3 modifications on chromatin around the TSSs, as is seen in established LCLs; however this has not yet been formally demonstrated in newly infected cells.

## CONCLUDING REMARKS

Through the combined action of EBNA3C and EBNA3A and their interaction with the cellular PcG protein system, EBV has evolved a very effective countermeasure to OSR/OIS that appears to be critical in its normal life cycle to establish a latent infection and therefore initiate long-term persistence in B cells. *In vitro* this mechanism neatly overcomes a major early obstacle to cellular “immortalization,” making EBV one of the most potent transforming/immortalizing biological agents to have been identified. By utilizing an epigenetic mode of gene regulation to tackle the problem of OSR/OIS, key target ts genes including *INK4a* and *BCL2L11/BIM* are repressed not only in the infected cells, but also in their progeny; furthermore the genes become particularly predisposed to complete silencing by DNA modification. It is self-evident – since EBV stably ablates at least two major barriers to oncogenic transformation – that this will substantially increase the likelihood of EBV-infected B cells undergoing additional genetic and/or epigenetic changes leading to cancer (discussed further in Thorley-Lawson and Allday, 2008; Allday, 2009; Skalska et al., 2010; Paschos et al., 2012). This manipulation of the PcG system to specifically regulate key tumor suppressor genes in B cells makes EBV – to our knowledge – unique among tumor viruses. Now the challenges are to provide complete biochemical descriptions of how the EBNA3 proteins interact with PcG complexes and – employing genome-wide screens such as ChIP-seq – determine the extent of polycomb-mediated epigenetic reprogramming of B cells by EBV.

## Conflict of Interest Statement

The author declares that the research was conducted in the absence of any commercial or financial relationships that could be construed as a potential conflict of interest.
